# Recent Progress towards Novel EV71 Anti-Therapeutics and Vaccines

**DOI:** 10.3390/v7122949

**Published:** 2015-12-08

**Authors:** Qingyong Ng, Fang He, Jimmy Kwang

**Affiliations:** 1 Animal Health Biotechnology, Temasek Life Sciences Laboratory, 1 Research Link, National University of Singapore, Singapore 117604; qingyong@tll.org.sg; 2 College of Animal Sciences, Zhejiang University, Yuhangtang Road 866, Hangzhou 310013, China; 3 Department of Microbiology Faculty of Medicine, National University of Singapore, Singapore 117604

**Keywords:** enterovirus 71, vaccines, anti-therapeutics, human intravenous immunoglobulin, baculovirus, monoclonal antibodies, inactivated vaccines

## Abstract

Enterovirus 71 (EV71) is a group of viruses that belongs to the *Picornaviridae* family, which also includes viruses such as polioviruses. EV71, together with coxsackieviruses, is widely known for its association with Hand Foot Mouth Disease (HFMD), which generally affects children age five and below. Besides HFMD, EV71 can also trigger more severe and life-threatening neurological conditions such as encephalitis. Considering the lack of a vaccine and antiviral drug against EV71, together with the increasing spread of these viruses, the development of such drugs and vaccines becomes the top priority in protecting our younger generations. This article, hence, reviews some of the recent progress in the formulations of anti-therapeutics and vaccine generation for EV71, covering (i) inactivated vaccines; (ii) baculovirus-expressed vaccines against EV71; (iii) human intravenous immunoglobulin (IVIg) treatment; and (iv) the use of monoclonal antibody therapy as a prevention and treatment for EV71 infections.

## 1. Introduction

Enterovirus 71 (EV71) is a group of viruses that belongs to the *Picornaviridae* family, which includes coxsackieviruses, polioviruses, echoviruses and rhinoviruses. EV71 has a size of approximately 30 nm, and like other members of *Picornaviridae*, it is a non-enveloped, positive, single-stranded RNA virus. Being a non-enveloped virus, its genomic RNA is packaged within the capsid that is made up of 60 copies of the pentameric subunits composed of the four structural proteins, namely VP1, VP2, VP3 and VP4, with VP1 to VP3 exposed on the exterior of the capsid, and VP4 located internally [1,2,3]. Besides the four structural proteins mentioned, the ~7.4 kb viral RNA also encodes for non-structural proteins such as RNA-dependent RNA polymerase, as well as proteases that are required for the autocatalytic processing of the viral proteins [4].

EV71 is classified into three major genotypes (A–C), which are further subdivided into 11 subgenotypes, namely A, B1 to B5, and C1 to C5. Such classification of EV71 into its subgenotypes is dependent on its VP1 sequence variations [5].

Like coxsackieviruses, EV71 is often known for its manifestation as Hand Foot Mouth Disease (HFMD), which affects mainly children of age five and below [6]. Unlike coxsackieviruses, EV71 infections are also more occasionally associated with severe neurological diseases such as poliomyelitis-like paralysis and aseptic meningitis and encephalitis, which could cause severe medical complications, as well as putting the lives of the infected individuals at stake [6].

Ever since its first isolation in the 1960s, EV71 has caused numerous epidemic outbreaks throughout world [7]. Within the last decade, large outbreaks mainly manifested in the Asia-Pacific regions, with Singapore, Malaysia, Taiwan, Indonesia and China frequently impacted [5,8,9,10]. Surges in the number of cases of EV71 infections have been observed in recent years; for instance, there was a 3.5-fold increment in the number of Hand Foot Mouth Disease (HFMD) cases observed in Singapore just between 2001 and 2006 [11]. Additionally, according to World Health Organization (WHO), a one-million-case increase in the number of HFMD cases has been observed in China alone between 2011 and 2014 [12].

Presently, there is no commercial antiviral drug or vaccine against EV71 available to the masses. The prevention strategies adopted by many authorities generally rely on maintaining good personal hygiene and quarantining infected individuals, so as to curb the spread of the virus. This lack of an anti-viral drug and vaccine against EV71 is largely due to two facts; (i) EV71 is a virus isolated only approximately 40 years ago, and many aspects of this virus remain to be elucidated; and (ii) generally EV71 infections are non-lethal and self-limiting, and this leads to a lack of research attention [7]. However, as time passes, more reports regarding the ability of EV71 to endanger the lives of infected individuals are surfacing, leading to the realization of the urgent need to have the tools for mankind to fight against these viruses.

This article, hence, focuses on the recent progressions of some developing vaccines and treatments against EV71, which include (i) inactivated vaccines, which are currently in the third phase of the clinical trials; (ii) baculovirus-expressed vaccines against EV71; (iii) human intravenous immunoglobulin (IVIg) treatment; and (iv) the use of monoclonal antibody therapy as a prevention and treatment for EV71 infections.

## 2. Inactivated Vaccines

Vaccines have always been considered as one of the most effective preventions against various types of infections, ranging from bacterial to viral infections. In fact, their effectiveness in the prevention of infectious diseases could be clearly observed in the eradication of smallpox, declared officially in 1976 by WHO, just two centuries after Edward Jenner showed the prevention of smallpox infection by inoculating cowpox into a person [13,14].

Similar to other viruses, the development of vaccines is seen as one of the top-most priorities in the fight against EV71. Various types of vaccines have been proposed and are currently under development. Among these vaccines, inactivated vaccines are the nearest to certification for market release [15].

Presently, there are three inactivated vaccines against EV71 that have completed their phase III clinical trials, and currently waiting for the approval by the appropriate authorities. Each of these vaccines was developed by individual organizations, namely (i) Sinovac; (ii) Vigoo; and (iii) the Kunming Institute, with a similar goal, which is to provide good acquired immunity when inoculated into humans [16]. All three vaccine strains were of the C4 subgenotype and were reported to have good safety and vaccine efficacy in their phase III clinical trials. It has been shown that all three vaccines were proven to prevent more than 80% of EV71-associated diseases in their respective trials where C4 was the prevalent subgenotype spreading [17,18,19]. In addition, all three vaccines were reported to induce reasonably good neutralizing antibody response in their clinical trials. At day 56 post-vaccinations, the inactivated vaccines of Vigoo and Sinovac showed a GMT (Geometric Mean Titer) of 28.4 times and 22.1 times higher than that of pre-vaccination, respectively [17,19]. No GMT of pre-vaccination was given in Kunming’s inactivated vaccine trial. Nonetheless, a GMT titer of 106 was reported, as compared to the 325.3 and 165.8 for Vigoo and Sinovac, respectively [17,18,19]. GMTs for all three vaccines dipped between the 56 days to eight months post-vaccination, but remained relatively constant (with a GMT above 85 for all three vaccines) thereafter [17,18,19]. These inactivated vaccines, hence, have been shown to be effective in providing protection against EV71 infections during their clinical trials.

Even though the trial results suggested that the inactivated vaccines are effective against EV71 infections, their effectiveness may be limited when deployed worldwide. The reason lies in the fact that each of these vaccines was derived from a particular seed strain of EV71, and research has shown that cross-immunity and cross-neutralization between genogroups may be limited when subjects are vaccinated using a particular strain [16,20,21]. Additionally, with the fact that only three known epitopes are capable of inducing neutralizing antibodies, with two of these epitopes found to stimulate the production of universal neutralizing antibodies, identified until date, may somehow suggest that such highly conserved antigenic sides among all the EV71 genogroups may be rather rare [22,23]. This, in turn, may explain the limited cross-immunity and cross-neutralization observed by the various research groups. The effectiveness of these inactivated vaccines is, therefore, questionable, especially when used in regions where other EV71 subgenotypes, different from the seed strain, are the main prevalent pathogens spreading in the communities.

Additionally, even though the highly conserved epitopes are found to be present in all the subgenotypes of EV71 based on sequence alignment, the generation of sufficient cross-immunity among all subgenogroups with just one inactivated strain is not easy [24]. This is supported by the results of various research teams, where the anti-EV71 sera, derived from the vaccination with a particular strain of EV71, are unable to provide cross-protection against all subgenogroups [25,26]. One plausible reason for this phenomenon could be the sequence variations that lie outside of these highly conserved regions, causing the folding of the viral proteins to be different among the subgenogroups. With the viral proteins being folded differently, the degree of exposure of these neutralizing epitopes is different among the subgenogroups. This, hence, would lead to the differences in the amount of universal neutralizing antibodies produced, translating to the different neutralizing capacity observed.

Making the issue regarding the existence of only few universal epitopes worse, some of these precious epitopes were lost during the production process, since formaldehyde destroys the native structure of the proteins [27]. The effectiveness of EV71 inducing cross-immunity and cross-neutralization among all subgenogroups may be further limited.

Another issue with inactivated vaccines is the possibility of triggering antibody-dependent enhancement (ADE) events upon the immunization of a healthy individual. ADE occurs when non-neutralizing antibodies bind to the virus particles, enhancing the virus’s entry into cells [28,29]. Such enhancement is the result of the interaction between the Fc region of the non-neutralizing antibodies and the Fc receptors present on certain cell types, particularly the THP-1 cells [28,29]. By easing the entry of the virus, this leads to higher rates of cell infections, which could have devastating impacts on the patients’ health.

Two research groups have shown and proven ADE occurrence for EV71 infections [29,30,31]. With 11 subgenotypes, the antibodies generated against one inactivated vaccine, belonging to one subgenotype, upon immunization may be able to bind to, but not neutralize, the other 10 subgenotypes. In the event when another subgenotype replaces the prevalent one that is responsible for most EV71 infections initially, the outcome could be a massive outbreak of life-threatening EV71 infections among children, who have previously recovered from such infections. Therefore, the use of EV71-inactivated vaccines should be further evaluated for their ability to trigger ADE events prior to their market release and usage.

With the route of administration being subcutaneous or intramuscular, inactivated vaccines generally induce systemic immune response, which leads to the production of IgM and IgG, with little IgA [32]. With EV71 transmitted via the feco-oral route, the lack of IgA production with the use of inactivated vaccines may, thus, pose another serious concern with inactivated vaccines. Hence, oral immunization may be a better alternative since such a route of immunization induces both mucosal and systemic immune responses [33].

## 3. Baculovirus-Expressed Vaccines

Baculoviruses are a group of insect-specific pathogens, with each species and/or strain targeting a very limited host range [34]. A particular strain of this virus, namely Baculovirus *Autographa californica* nuclear polyhedrosis virus (AcNPV), capable of infecting a broad range of lepidopteran hosts, was isolated, and eventually formed the basis for modern day recombinant baculovirus vectors, which are widely used for protein expression [34,35,36].

The baculovirus expression system (BVES) has been widely exploited in modern-day research for protein expression due to its superiority over the traditional expression systems, in particular the *E. coli* expression system [37]. Such superiority of BVES could be accounted for by a few traits, with one of the most significant being the ability express eukaryotic proteins in their native conformation, something not possible with the *E. coli* expression system [37,38,39]. As BVES relies on eukaryotic insect cells for protein expression, the expressed proteins could be post-translationally modified, such as being phosphorylated and glycosylated [34]. In contrast, being prokaryotic in nature, the *E. coli* expression system lacks such modifications. This, hence, explains why BVES, but not the *E. coli* expression system, is capable of producing eukaryotic proteins in their native conformation.

The ability of expressing proteins in their native form can be observed in the expression of influenza hemagglutinin (HA) protein using BVES. HAs are glycoproteins found on the surface of influenza viruses. They bind to the sialic acid found on cells, such as erythrocytes, and such binding causes the erythrocytes to clump together, *i.e.*, hemagglutinates. In order for hemagglutination to occur, the HA must be in the right conformation, and it has been shown that *E. coli*-expressed HAs formed inclusion bodies and failed to induce hemagglutination when mixed with red blood cells [40]. In contrast, various researchers have successfully expressed HAs that were capable of inducing hemagglutination using BVES [41,42,43]. Hence, this proves the ability of BVES to express (viral/mammalian) proteins in their native configurations. In addition, BEVS also allows the formation of multi-protein subunit complexes, which is not observed in the *E. coli* expression system [44,45]. This is especially important when expressing larger proteins with tertiary configurations, common in mammalian-expressed proteins.

Expression of proteins in their native configurations is exceptionally advantageous when it comes to vaccine production as this would allow the retention of not only the rightly exposed linear epitopes, but also those conformational ones. This, hence, makes BVES a very attractive option for vaccine production.

In general, there are several advantages of baculovirus-expressed vaccines, such as baculovirus-expressed virus-like particles (VLPs) and baculovirus surface display, over inactivated vaccines. One advantage of baculovirus-expressed vaccines is the complete elimination of any fear that may arise from the incomplete inactivation of the highly pathogenic viruses during the manufacturing process of inactivated vaccines, which is a concern that inactivated vaccines hold. Such extra peace of mind is attributed to two facts, namely (i) no (complete) EV71 virus genomic material is present in baculovirus-expressed vaccines; and (ii) baculoviruses are harmless, largely owing to their non-replicable nature in mammalian cells [45,46,47].

Besides the extra peace of mind that baculovirus-expressed vaccines have over inactivated vaccines, no large-scale bio-containments are required during the manufacturing of baculovirus-expressed vaccines, since baculovirus does not cause harm to human beings and its handling only requires biosafety class 1 practices [20]. In contrast, large bio-containments are required for inactivated vaccine production, especially when handling the pathogens before and during the inactivation process. As no bio-containments or extra safety equipment are required, this would translate to a much lower production cost for baculovirus-expressed vaccines as compared to the cost of producing inactivated vaccines.

As mentioned earlier, the formaldehyde used in the inactivation process would destroy some of the native conformation of the viral structural proteins [27]. This is supported by experimental research where sera from mice immunized with formalin-inactivated EV71 had lower neutralizing titers than Binary ethyleneimine (BEI)-inactivated ones [48]. The use of formaldehyde for inactivation would, hence, reduce the efficacy of the vaccine in eliciting neutralizing antibodies upon inoculating into healthy individuals. On the contrary, baculovirus-expressed vaccines would not face this issue since no inactivation would be required, and this is clearly another advantage they have over inactivated vaccines.

### 3.1. Baculovirus-Expressed Virus-Like Particles (VLPs)

With the abilities of expressing proteins in their native forms and the formation of multi-protein complexes, BVES permits the formation of virus-like particle (VLPs) [34]. Virus-like particles (VLPs) are particles that hold strong structural resemblance to a particular virus-of-interest [49]. These particles, however, lack the viral genomic material as well as most, if not all, of the viral non-structural proteins [49]. These particles, hence, are unable to replicate like the normal virus, posing no harm to any individuals. Due to their harmless nature and their structure resemblance to the native virus, these particles are believed to be a better substitute for the inactivated virus vaccines.

Due to VLPs having similar efficacy in eliciting both innate and cell-mediated immune responses as the inactivated vaccines, as well as their harmless nature, there is no doubt that many research groups are interested in designing baculovirus-expressed VLP vaccines [50]. Currently, several research teams have successfully expressed EV71-VLPs using BVES. Briefly, these groups generally cloned EV71 P1 and 3CD genetic sequences into the baculovirus vectors, which are subsequently transfected into an insect cell line, such as SF9 or Hi Five cells [51,52,53]. Upon transfection, these cells would express the P1 and 3CD proteins. The expressed 3CD proteins will then cleave the P1 proteins accordingly into the individual matured viral subunits, namely VP1 to VP4, which would eventually self-assemble into the virus-like particles, which will be used for vaccinations [54].

In a study, it was found that the purified EV71 baculovirus-expressed VLPs are capable of inducing a neutralizing titer of 2^12.3^, which is statistically similar to the titer of 2^12^ when inactivated EV71 vaccines are used [51]. This, thereby, further shows that VLPs are a viable alternative to inactivated vaccines.

Theoretically, baculovirus-expressed VLP vaccines provide an excellent alternative over inactivated vaccines. However, baculovirus-expressed VLPs have rather low yields, a major issue that impedes their utilization for mass production in real life. Such low yields would translate to being economically non-feasible for large-scale production [51]. There are many efforts in trying to improve the yields of baculovirus-expressed VLPs, such as placing both 3CD and P1 into one recombinant vector, as well as using different promoters for 3CD and P1 to obtain an optimal 3CD:P1 protein ratio [45,51]. In addition, a research group has recently employed the use of suspension Hi Five cells, instead of SF9 cells, in hopes of increasing the overall yield [53]. Improvements in the yield have been observed with the implementations of these efforts, but there still remains a big gap for baculovirus-expressed VLPs to be termed economically viable for mass production.

### 3.2. Baculovirus Surface Display

With low yield plaguing baculovirus-expressed VLPs, baculovirus surface display vaccines may be a better option for vaccine development. Baculovirus surface display vaccines are based on the expression of the desired antigenic proteins on the surface of the baculovirus envelope. Traditionally, such expression is made possible by splicing the sequence of the desired proteins with that of the baculovirus GP64 proteins [45,55]. As GP64 proteins would be incorporated into the membrane of the baculovirus during the budding process, the desired antigenic proteins, which are fused with GP64, would be displayed on the surface of the matured baculovirus. Upon inoculating into a healthy individual, the immune system would recognize and target these antigenic proteins on the baculovirus surface display, leading to the generation of immune protection against the EV71 virus.

**Figure 1:**
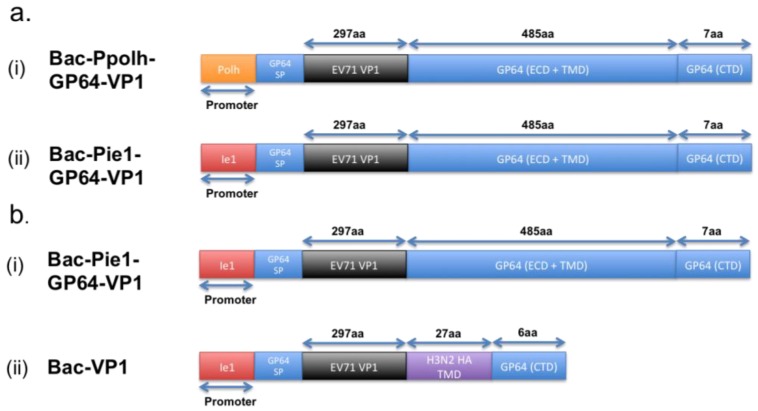
Schematic illustration of the differences between the variants for the GP64-VP1 expression cassettes. (a) Differences between the two initial GP64-VP1 vectors produced by Meng *et al.* [21], with (a-i) having a conventional Polh promoter and (a-ii) having a non-conventional ie1 promoter. (b) Differences between the Bac-Pie1-GP64-VP1 vector and a greatly shortened Bac-VP1 vector by Premanand *et al.* [48]. The latter has the GP64 ECD and TMD domains replaced with a 27aa H3N2 haemagglutinin TM domain. SP: Signal Peptide, ECD: Extracellular Domain, TMD: Transmembrane Domain, CTD: Cytoplasmic Domain.

A research group has previously expressed the EV71 VP1 proteins on the baculovirus and evaluated the use of such recombinant baculovirus as a vaccine. In their study, the team constructed two different recombinant baculovirus vectors, each harboring the VP1 protein sequence fused with that of the GP64 protein. The difference between the two vectors, shown in Figure 1a, lies in the promoter used for the regulation of the respective recombinant GP64-VP1 expression cassettes [21]. One of the two vectors uses the traditional polyhedrin (polh) promoter, whereas the other has the non-conventional ie1 promoter. The polh promoter is traditionally used, as it is well known for its high expression rate [21,34]. However, as hypothesized and proven by the research group, using the ie1 promoter instead of the polh promoter would lead to an increased incorporation of VP1 proteins on the surface of the resultant recombinant baculovirus, as the ie1 is an immediate-early promoter whereas the polh is a late promoter. With the increase of VP1 incorporation, the Bac-Pie1-GP64-VP1 elicited greater neutralizing titers than Bac-Ppolh-GP64-VP1 [21]. Supportively, the neutralizing titer, when Bac-Pie1-GP64-VP1 baculovirus was used to vaccinate mice, was just two times lower than that when heat-inactivated EV71 was used [21]. This, hence, shows the feasibility of using surface display vaccines.

However, comparing the baculovirus-expressed EV71 VLPs with Bac-Pie1-GP64-VP1 surface display, the current EV71-VLPs induce a higher amount of neutralizing antibodies than Bac-Pie1-GP64-VP1. Based on other studies by the same research group, this lower immunogenicity for Pie1-GP64-VP1 surface display could be attributed to the lower recombinant baculovirus titer [48]. Such lower titer is believed to be a result of the presence of the large GP64-VP1 extracellular domain that is likely to interfere with the budding of baculovirus from the host cells. By replacing the GP64 transmembrane and extracellular domains with just a 27-amino-acid-long HA transmembrane domain, the team showed an increase in sera-neutralizing titers by four to eight times [48]. In addition, the sera-neutralizing titers are higher than that induced by formalin-inactivated EV71. Although the reduction of the overall size of the extracellular domain could improve the induction of a higher neutralizing titer upon immunization, the titer remains to be lower than that when BEI-inactivated EV71 virus, which retains all linear and conformational epitopes, is used for vaccination [48]. This, thus, implies that the larger GP64-VP1 extracellular domain is not the entire cause for this lower titer.

The lower immunogenicity of Pie1-GP64-VP1 surface display could be caused by the fact that only VP1s are displayed on the recombinant baculovirus whereas all four EV71 structural proteins are present, not to forget in the right configuration, in VLPs. There are logically more neutralizing epitopes present in VLPs. Scientifically, this is backed up by the known neutralizing epitopes, summarized in Table 1, that have been identified over years by various research groups. In Table 1, it is clearly shown that besides VP1, VP2 and VP3 also contain neutralizing epitopes.

**Table 1:** Summary of reported EV71 neutralizing antibodies.

Mab Name	Derived from EV71 Genotype	Target	Epitope Type	Epitope Sequence	Remarks
MAB51 [22]	NUH0083/subgenotype B5	VP1	Linear	KQEKD	Able to neutralize all 11 subgenotypes of EV71. Universal neutralizing antibody.
10D3 [23]	5865/SIN/000009/subgenotype B4	VP3	Conformational	Amino acid position 59, 62 and 67 formed the highly conserved knob	Able to neutralize all 11 subgenotypes of EV71. Universal neutralizing antibody.
4E8 [24]	EV71 Hn2 strain/subgenotype C4	VP1	Linear	SSKSEYSLVI (aa 240–250); RIYMRMKHVR (aa 250–260)	Neutralization only tested on Hn2 Strain. RIYMRMKHVR epitope is highly conserved among 11 EV71 subgenotypes. SSKSEYSLVI epitope is not highly conserved.
4C6 [24]	EV71 Hn2 strain/subgenotype C4	VP1	Linear	SSKSEYSLVI (aa 240–250); RIYMRMKHVR (aa 250–260)	Neutralization only tested on Hn2 Strain. RIYMRMKHVR epitope is highly conserved among 11 EV71 subgenotypes. SSKSEYSLVI epitope is not highly conserved.
MAb979 [56]	EV71/subgenotype C2	VP2	Linear	Amino acids 136–150, encompasses the exact epitope EDSHP	Neutralizes B4, B5, C2 with cross-reactivity with CVA16.
MA28-7 [57]	EV71 strain 1095/subgenotype C2	VP1	Conformational	VP1-145 and residues that map to the positively charged patches (VP1-98, VP1-242, and VP1-244)	Neutralizes EV71 subgenotype A, B1, B3, B4 and C2. Neutralizes only specific strains of EV71 that have a conserved glycine at amino acid VP1-145, a surface-exposed residue that maps to the five-fold vertex and that has been implicated in receptor binding.
BB1A5 [58]	EV71 strain 52-3/subgenotype C4	VP2	Linear	136-155 (Thr (T141A), Glu (E142A), Ser (S144A) and His (H145A) being the most critical points)	Able to neutralize B3, B4, C2, C4 and C5 subgenogroups.
H3B10 [59]	Not known	VP1	Linear	KQEK (aa 208–222)	Shown capable of neutralizing C2, C4, C5, B4 and B5 subgenogroups. Broad-neutralizing monoclonal antibodies.
K8G2 [59]	Not known	VP1	Linear	KQEK (aa 208–222)	Shown capable of neutralizing C2, C4, C5, B4 and B5 subgenogroups. Broad-neutralizing monoclonal antibodies.
2G8 [60]	EV71 strain AH/08/0 /subgenotype C4	VP1	Linear	YPTFGEHKQEKDLEYC	Shown capable of neutralizing C4 subgenogroup. Ala substitutions at Y208, T210, G212, K215, K218, L220, E221, and Y222 could completely abolish or significantly reduce the binding reactivity.
22A12 [61]	EV71 strain 1095/Shiga /subgenotype C2	VP1	Linear/Conformational	YPTFGEHKQEKDLEYC (Amino acid residues 211 to 217 of the GH loop are disordered in the procapsid but form a structured loop in the infectious virus. 22A12 recognizes the disordered loop only)	Unable to neutralize infectious virus (native virus). Able to bind to procasid, which protects the live virus from neutralizing antibodies. When being binded to 22A12, the protective shield is lost, hence increasing the neutralizing ability of other antibodies.
D2 [62]	Yeast-produced virus-like particles	VP1	Linear	Not known	Produced by immunizing with yeast-produced virus-like particles rather than whole virus.
G12 [62]	Yeast-produced virus-like particles	VP1	Linear	Not known	Produced by immunizing with yeast-produced virus-like particles rather than whole virus.
Mab 22 [63]	EV71 Tainan/4643/98/C2	VP1	Conformational	Not known	With neutralization capabilities but no data on the subgenogroups tested.
Mab 24 [63]	EV71 Tainan/4643/98/C2	VP1	Conformational	Not known	With neutralization capabilities but no data on the subgenogroups tested.

A suggested improvement that could be made to the current baculovirus surface display would be to ideally co-express and co-display of all three VPs on the one recombinant baculovirus. By incorporating VP1 to VP3 on the same baculovirus would most likely increase the immunogenicity of the end product since most, if not all, EV71-neutralizing epitopes would be present. By increasing the immunogenicity of the baculovirus surface display, this would definitely make such a vaccine the most promising tool against EV71 infections.

Baculovirus surface display vaccines could be used orally to effectively induce both mucosal and systemic immunity as previously reported by Mookkan *et al.* [64] and Tao *et al.* [21]. Similar to the HA of influenza virus, the VP1 of EV71 is responsible for the binding of the virus to the epithelial cells of the intestine [21]. This would probably induce the mucosal immune system, thus leading to the production of the desirable IgA upon vaccinations. Baculovirus surface display vaccines, therefore, serve as the better form of vaccine candidates.

Moreover, unlike VLPs and inactivated vaccines, no adjuvant may be required for baculovirus surface display vaccines when used as vector vaccines. The potential of baculovirus surface display vaccines as vector vaccines could be accounted for by three properties of such vaccines.

Firstly, live wild-type baculoviruses alone are proven to induce immune responses, both innate and cell-mediated immunity, upon their inoculation into the body [64,65,66,67,68]. Hence, displaying the antigens on the baculovirus surface and using such recombinant baculoviruses live may reduce the need for adjuvants. Secondly, although baculoviruses are unable to replicate within mammalian host cells, they are capable of transducing them [45]. Lastly, the Ie1 promoter used to regulate the expression of foreign genes in the baculovirus surface display is an active promoter not just in insect cells, but also in mammalian cells [69].

Taking the latter two properties together, the transduced mammalian cells, especially those antigen-presenting cells (APC), will express the foreign proteins. Such expression of foreign proteins within the APCs would be further processed and later cross-presented by Major Histocompatibility Complex (MHC) class II on the cell surface of these APCs [70,71]. This presentation of the MHC: Antigen complexes would then lead to the activation of CD4^+^ T-cells, which subsequently secrete the necessary cytokines that will further activate the (naïve) B-cells (one of the APCs), leading to their proliferation, and later isotype switching and affinity maturation [72,73]. These events would lead to the expression and secretion of IgM initially and, eventually, IgG antibodies that target the particular pathogen-of-interest.

Compared to the inactivated and VLP vaccines, baculovirus surface display vaccines, when used as vector vaccines, will induce more intense MHC: antigen presentations as the antigens that will be processed and used for such presentations are not only those introduced during immunization, but also those expressed by the transduced cells. With a more intensive MHC: antigen presentation, a greater immune response could be induced, hence reducing the need for adjuvants.

Besides MHC class II presentations, the expressed antigens within the transduced cells would also lead to the MHC class I presentations, which activate the CD8^+^ cytotoxic T-cell (CTL) response [74]. The activation of the CTL response would lead to the effective killing and removal of pathogen-infected cells [74], implying that such vaccines are also effective for therapeutic treatments besides being used as prophylactics, a unique advantage of baculovirus surface display over inactivated viruses and VLPs as vaccines [75]. Taken together, the potential of using baculovirus surface display vaccines as vector vaccines would induce a good level of immunity even without the use of adjuvants.

## 4. Intravenous Immunoglobulin (IVIg) Treatment

Vaccinations are only suitable for individuals with immune systems capable of raising an immune response against the foreign targets/particles. This fundamental criterion for successful vaccinations keeps immunocompromised people, such as X-Linked Agammaglobulinemia (XLA)-and Common Variable Immune Deficiency-affected individuals, unprotected even if an EV71 vaccine is made available on the market, since their bodies are unable to produce sufficient and/or functional immunoglobulins.

Intravenous immunoglobulin (IVIg) treatment thus becomes a great option in providing protection for these groups of people [76]. IVIg is the intravenous injection of polyvalent IgG antibodies that are purified from blood plasma collectively pooled from thousands of donors [76]. In fact, such methods have been constantly used to provide passive immune protection against general infections for immunocompromised individuals [76]. It has also widely used in certain autoimmune diseases, such as Idiopathic Thrombocytopenic Purpura (ITP) and Kawasaki disease [76,77]. Additionally, clinical administrations of IVIg in confirmed EV71-infected individuals, who are in a dire situation, led to changes in both pro- and anti-inflammatory cytokine levels, which are responsible for the rapid relief of EV71 symptoms, suggesting its therapeutic effect [78,79]. Moreover, the IVIg products contain neutralizing antibodies, which may provide individuals with the necessary protection against EV71 [80,81]. IVIg thus may be a wonderful tool to provide both therapeutic and prophylactic treatments against EV71.

Clearly, the neutralizing antibodies present within the IVIg product are responsible for the prophylactic protection [31,81]. On the other hand, the mechanism for the therapeutic effect provided by IVIg is still yet to be fully elucidated. Several theories have been proposed to explain this therapeutic effect observed. Some of these theories include the presence of EV71-neutralizing antibodies, the blockage of the Fc receptors on macrophages by the polyvalent IgGs, the presence of anti-autoimmune antibodies that suppress cytokines and chemokines, as well as the presence of idiotypic antibodies that suppress autoimmune antibodies [31,81,82,83].

The use of IVIg does have some concerns. IVIg is technically polyvalent IgGs that were pulled collectively from thousands of healthy individuals, as mentioned earlier. In addition, it was found that different preparation methods would yield IVIg products of different composition with different biological responses upon administration [84,85]. Hence, different brands and batches of such products may have different degrees of effectiveness against EV71 prevention or treatment since the pooled serum content is different each time. Therefore, there is likely to be a lack of consistency in its therapeutic and prophylactic efficacy.

Like any blood-derived products, IVIg holds the concern of containing blood-borne pathogens, such as Human Immunodeficiency Virus (HIV), Hepatitis B and Hepatitis C [86]. Stringent blood screening and even pathogen inactivation are, thus, necessary prior to the utilization of the collected blood for IVIg production. Such screening and inactivation procedures would definitely increase the cost of production, which would translate to a higher selling price, keeping these products out of reach from people living in developing countries. Moreover, chances of false negative results when performing the blood screening are still present. Hence, there are reservations with the use of IVIg for any treatment, including that for EV71.

The use of IVIg may also lead to ADE events. One study has shown that an insufficient concentration of IVIg products used enhances EV71 infection, as compared to when no such products were administered [30]. This demonstrates the possibility of ADE when IVIg products are administered. Another study has suggested that IgG3 generally has little neutralizing effect against EV71 and this isotype contributed to ADE instead [31]. The usage of IVIg in EV71 prevention or treatment may, thus, lead to more harm than good since the IVIg products contain many such non-neutralizing IgG3 antibodies. More intensive studies are, therefore, required to prove that IVIg is safe for its usage against EV71 infections before such a product is made widely available to the public.

## 5. Monoclonal Antibody Therapy against EV71

Similar to IVIg, the use of neutralizing antibodies is considered a form of passive immunity, since the immunity comes from the antibodies being introduced into the subjects, *i.e.*, not natively produced. Monoclonal antibody therapy could thus serve as the alternative option to IVIg for providing protection against specific pathogens, especially for the immunocompromised. Ideally, the monoclonal antibody valuable for such therapy should not only have high neutralizing efficacy against its targeting pathogens, but also a broad neutralizing spectrum which covers most, if not all, of the subgenotypes of a particular species of virus.

Over the years, a number of EV71-neutralizing antibodies have been generated and reported by various research groups around the world. These reported antibodies, together with the epitopes they recognize, have been summarized in Table 1. As shown in Table 1, to date, two antibodies with universal neutralizing capabilities, *i.e.*, that are able to neutralize all 11 subgenotypes of EV71, have been reported, and these include (i) Mab51 and (ii) 10D3, with both of them as IgM isotypes [22,23]. One key attribute to their universal neutralizing capabilities is their ability to recognize highly conserved epitopes that are present in all EV71 subgenotypes. Mab51 recognizes a highly conserved linear epitope, with a amino acid sequence of KEQK, found in VP1 of EV71, whereas 10D3 recognizes a conformational epitope that lies on a highly conserved knob region found in VP3 [22,23]. It was reported that both antibodies have high neutralizing efficacy *in vitro*, where Mab51 and 10D3 were found to have a neutralization titer of 10^5^ (against all subgenotypes) and 10^6^ to 10^8^, respectively [22,23]. In addition, both antibodies have also been shown to provide good prophylactic protection against a lethal dose of EV71 in mice. In two separate studies, a single dose (10 ug/g body weight) of Mab51 was sufficient to protect mice that were challenged with a lethal dosage of EV71 [22]. Similarly, 10D3 provided comparable protection *in vivo*, but at a higher dosage (10 mg/g) [23]. These results suggested the potential usefulness of these monoclonal antibodies as effective EV71 prophylactics.

The neutralizing monoclonal antibodies reported to date, including 10D3 and Mab51, against EV71 are all prophylactic in nature, without any therapeutic effects. This means that these antibodies can only be served as a preventive measure rather than for treatment usage. This, hence, would leave monoclonal antibody therapy rather useless in treating confirmed EV71-infected patients. Nonetheless, a newly isolated universal monoclonal antibody from our lab has been shown in a preliminary *in vivo* study (based on unpublished data) to protect EV71-infected mice both prophylactically and therapeutically. This, thus, proves not only the possibility of generating and isolating such highly valuable monoclonal antibodies, but also the possibility of using monoclonal antibodies as both prophylactics and therapeutics in the fight against the EV71 infections.

With ADE and product consistency being a concern for IVIg, the use of neutralizing monoclonal antibodies may serve as a better alternative than IVIg. The difference between IVIg and monoclonal antibody treatments lies in the content of the products. IVIg contains all kinds of polyvalent IgG antibodies, cytokines and chemokines, with little IgM and IgA, whereas in monoclonal antibodies treatment, only specific neutralizing antibodies are involved [76]. In other words, monoclonal antibodies are more specific and the risks of ADE events would be much lower. The production of monoclonal antibodies can also be easily scaled up with consistency since the hybridoma cell lines are immortal and stable [87]. In contrast, the IVIg product is derived from thousands of healthy individuals and the therapeutic and prophylactic efficacies between batches are bound to have differences.

Hybridomas that produce monoclonal antibodies are typically generated through the fusion of myeloma and mouse spleenocytes [88]. The antibodies produced would, therefore, be of mouse origin since both types of cells are derived from mice. Such antibodies would lead to immune rejection when injected into human bodies since antibodies are immunogenic in nature [89]. In addition, the murine antibodies are unable to elicit the appropriate downstream immune responses since the Fc receptors on immune cells, like those on macrophages, are unable to recognize the murine Fc regions [90,91].

In recent years, several technologies, in hopes of resolving this issue, have been developed. These methods include chimerizing and humanizing antibodies, using transgenic mice for human monoclonal antibody production, as well as immortalizing human memory B-cells derived from patients’ blood [92,93,94,95]. The latter two technologies are still not widely adopted in today’s context as they have their limitations. Using memory B-cells for monoclonal antibody production, for instance, is limited by the fact that it is impossible to put humans through the type of immunization regime performed during murine monoclonal antibody production [96]. As for the use of transgenic mice for monoclonal antibody production, since only a portion of genes coding for the human V-regions (both heavy and light) are knocked into the mice germline cells, the resulting mice have a much lower diversity of antibodies that can be produced [97,98]. This could, hence, explain the transgenic mice being a less robust system for antibody production than the traditional mouse strains used for mouse antibody production [99]. In addition, it was reported that such transgenic mice have issues regulating the expression of human immunoglobulin genes [100].

Before humanization comes to light, the chimerizing of antibodies is a way to reduce immunogenicity when injected into a human body. In chimerization, the Fab sequences of the murine antibodies are being spliced into templates containing the sequences for the human Fc region [101,102]. The resultant recombinant DNA will then be knocked into an antibody-producing cell-line for the generation of the chimeric antibodies, which contains the Fab region of murine origin and Fc region of human origin [103]. Since the majority of the immunogenic portions of the antibodies lie in the Fc region, chimeric antibodies have a much lesser immunogenicity compared to the fully murine ones [104]. However, even with a greatly reduced immunogenicity, the chances of immune rejection after being inoculated into humans remain a concern since these chimeric antibodies still contain reasonable portions belonging to murine origin [104].

Fortunately, with the advancement in antibody research, the humanization of murine antibodies is made available prior to passive immunization. The humanization of antibodies involves grafting only the three Complementarity Determining Regions (CDRs) region into a human antibody variant, and this greatly reduces, if not eliminates, the problem of immune rejection upon being injected into humans since the majority of the antibody structure is of human origin [103,104]. The feasibility of humanizing antibodies can be seen in the humanized monoclonal antibody research against H5N1 where the humanized antibody h8A8 retains all its neutralizing ability against the different H5 strains [105]. Additionally, the antibody-neutralizing titer remains comparable to the parental murine antibody [105]. Certainly, the humanization of neutralizing antibodies against EV71 is an attractive option for research and medical purposes, which is currently being pursued.

Even though humanization technologies have aided greatly in the reduction of the immunogenicity of murine monoclonal antibodies when used therapeutically, the choosing of the most appropriate Fc region (isotypes and allotypes) during the humanizing process still remains a problem. As briefly mentioned earlier, the Fc region determines the type of downstream effector functions/immune response that will be triggered, thus the selection of the Fc region to use in the humanization process is indeed important [106,107]. Currently, IgG1 is the most widely adopted Fc region for humanization due to its superiority in triggering both cell-mediated and complement effector functions. However, the mixed reviews on the hierarchy for the effectiveness of isotypes mediating Antibody-Dependent Cell-mediated Cytotoxicity (ADCC) events suggest that much is still unknown about the complete downstream events triggered by each isotype. Moreover, as mentioned earlier, IgG3 may contribute to ADE events in EV71 infections; hence, whether humanized IgG3 antibodies which are against EV71 would do more harm than good is still yet to be answered [31].

## 6. Conclusions

In conclusion, although inactivated vaccines are in the lead for reaching market release, baculovirus-expressed VLPs seem to be a better alternative to inactivated vaccines. This is largely owed to the advantages they have over inactivated vaccines, for instance better immunogenicity and fewer safety concerns. However, the cost of production for these VLPs remains an issue due to the low yield; hence, baculovirus surface display vaccines serve as the more attractive option. Nonetheless, more research aiming to improve the efficacy and efficiency of these baculovirus surface display vaccines should be performed. Needless to say, the baculovirus surface display holds the greatest potential in producing the cheapest yet reliable vaccine candidates. As vaccines could not provide protection for the immunocompromised, IVIg and monoclonal antibody therapy could be the solution for this group of individuals. However, considering the risks of ADE events and other safety concerns regarding the use of IVIg products, monoclonal antibody therapy is a more promising approach than IVIg. Nonetheless, with no therapeutic antibodies against all EV71 subgenotypes reported to date, IVIg could be considered as the only treatment available for EV71-infected patients who are in severe conditions. In general, more efforts are needed in researching and developing EV71 therapeutics and vaccines, and this should be a top priority to protect our young ones, who will eventually become the pillars of our society in the very near future.
